# Eyes wide shut: necessity and effect of adjunctive procedures after decompression surgery in patients with endocrine orbitopathy

**DOI:** 10.1186/s13005-021-00290-2

**Published:** 2021-09-15

**Authors:** Matthias Krause, Mohammad Kamal, Dirk Halama, Thomas Hierl, Ina Sterker, Rüdiger Zimmerer, Bernd Lethaus, Alexander K. Bartella

**Affiliations:** 1 Department of Oral- and Maxillofacial Surgery, Leipzig University, Liebigstraße 12, 04103 Leipzig, Germany; 2 Department of Surgical Sciences, Faculty of Dentistry, Kuwait University, Safat, Kuwait; 3 Department of Oral and Maxillofacial Surgery, Helios Vogtland Klinikum Plauen, Röntgenstraße 2, 08529 Plauen, Germany; 4 Department of Ophthalmology, Leipzig University, Liebigstraße 12, 04103 Leipzig, Germany

**Keywords:** Endocrine orbitopathy, Ocular surface area, Blepharoplasty surgery, Lid repositioning, Lid refinement, Digital facial-analysis tool, 3D photography

## Abstract

**Background:**

Orbital decompression surgery is frequently the last therapeutic measure in the surgical treatment of endocrine orbitopathy (EO). Additional rehabilitative and corrective surgical treatments are often used to improve the resulting eyelid stigmata, such as an increased lid aperture and scleral show. The aim of the study was to evaluate the effect of adjunctive surgical procedures after orbital decompression surgery in patients with EO.

**Methods:**

A total of 120 orbitae from 65 patients with EO from 2010 to 2020 at a tertiary care center in Germany were retrospectively evaluated. Ocular surface area (OSA) and vertical palpebral fissures were three-dimensionally analyzed at the following stages: presurgical decompression, postsurgical decompression, and post-adjunctive surgical procedures. For the analysis of vertical palpebral fissures, predefined vertical line distances were measured on the upper and lower lids in the central, medial, and lateral pupillary regions.

**Results:**

The initial OSA was 2,98 ± 0.85 cm^2^, and it decreased significantly after decompression surgery to 2.52 ± 0.62 cm^2^. After adjunct surgical procedures, OSA further decreased to 2,31 ± 0,55 cm^2^. Furthermore, a statistically significant reduction in all pupillary parameters was noted after each treatment step. More lid-lengthening procedures were performed on the lower lid than on the upper lid. Canthoplasty (*n* = 13) was the most frequently performed procedure during rehabilitation.

**Conclusion:**

Surgical decompression surgery improves OSA and leads to a significant reduction in lid aperture. Adjunctive surgical procedures, addressing the upper and lower lid, have a significant influence on the ongoing clinical course and contribute to a reduction in OSA.

## Introduction

Endocrine orbitopathy (EO), also known as Graves’ orbitopathy or thyroid eye disease, is the main extrathyroidal manifestation of Graves’ disease and is found in about 25% of patients at diagnosis, often as a mild and self-remitting condition. Severe forms, such as dysthyroid optic neuropathy (DON), affect 3–5% of patients. It presents as an ocular disease, causing esthetic disfigurement and functional deficits, and may lead to diplopia or even a loss of vision [1–3]. Rehabilitative surgery for functional and cosmetic rehabilitation (orbital decompression surgery, squint surgery, or eyelid surgery) is often required and has been traditionally performed in four stages: (1) orbital decompression, (2) extraocular muscle surgery, (3) correction of eyelid retraction, and (4) removal of excess tissue and fat [1, 4]. Upper and lower eyelid surgery is usually carried out as the final corrective surgical intervention and is considered an important step toward functional and esthetic eyelid rehabilitation [5]. However, surgical management is relatively complex, and preoperative prediction of changes in eyelid contour is difficult in many cases. Thus, it is important for the surgeon to have a wide range of options to treat potential problems after decompression surgery. Procedures such as eyelid lengthening, cartilage transplantation, blepharoplasty, and lipofilling may contribute to a more favorable long-term result and higher patient satisfaction. However, surgery depends not only on the array of possibilities but also on recognizing the right indication for each intervention in order to achieve successful results.

Thus, the aim of this study was to evaluate the necessity and effect of adjunctive post-decompression surgery in patients with EO. To achieve accurate and objective results, the effects of adjunctive procedures on the ocular surface area (OSA) and palpebral fissures were measured and evaluated using a three-dimensional (3D) stereophotogrammetric imaging system.

## Methods

This study was approved by the Local Ethics Committee (Eth-35/15) in accordance with the Declaration of Helsinki on Medical Protocol and Ethics. Informed consent was obtained. A retrospective evaluation of patient data with a diagnosis of EO in a tertiary care center from 2010 to 2020 was conducted for the study. A total of 123 orbitae from 67 patients were included.

Only patients who were surgically treated and who had previously undergone all conservative and causal treatment options to resolve EO but without success were included in the study. Two patients were excluded because they were diagnosed with malignant exophthalmos, and one of them presented with concurrent thyroid carcinoma in Graves’ disease. General demographic data, such as age, gender, and symptoms caused by EO, were recorded. Post-decompression surgical procedures were noted and correlated to the indication.

### Landmarking and 3D measurement

**Fig. 1:**
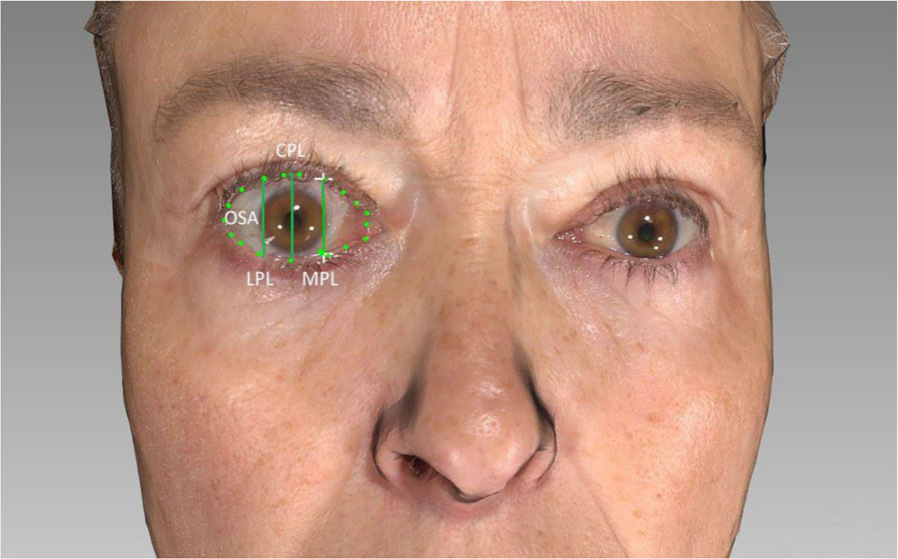
Measurement of ocular surface area (OSA) and lid aperture using FAT software. The scleral area and lid aperture were analyzed by placing landmarks. OSA ocular surface area; MPL medial pupillary line; CPL central pupillary line; LPL lateral pupillary line

**Fig. 2:**
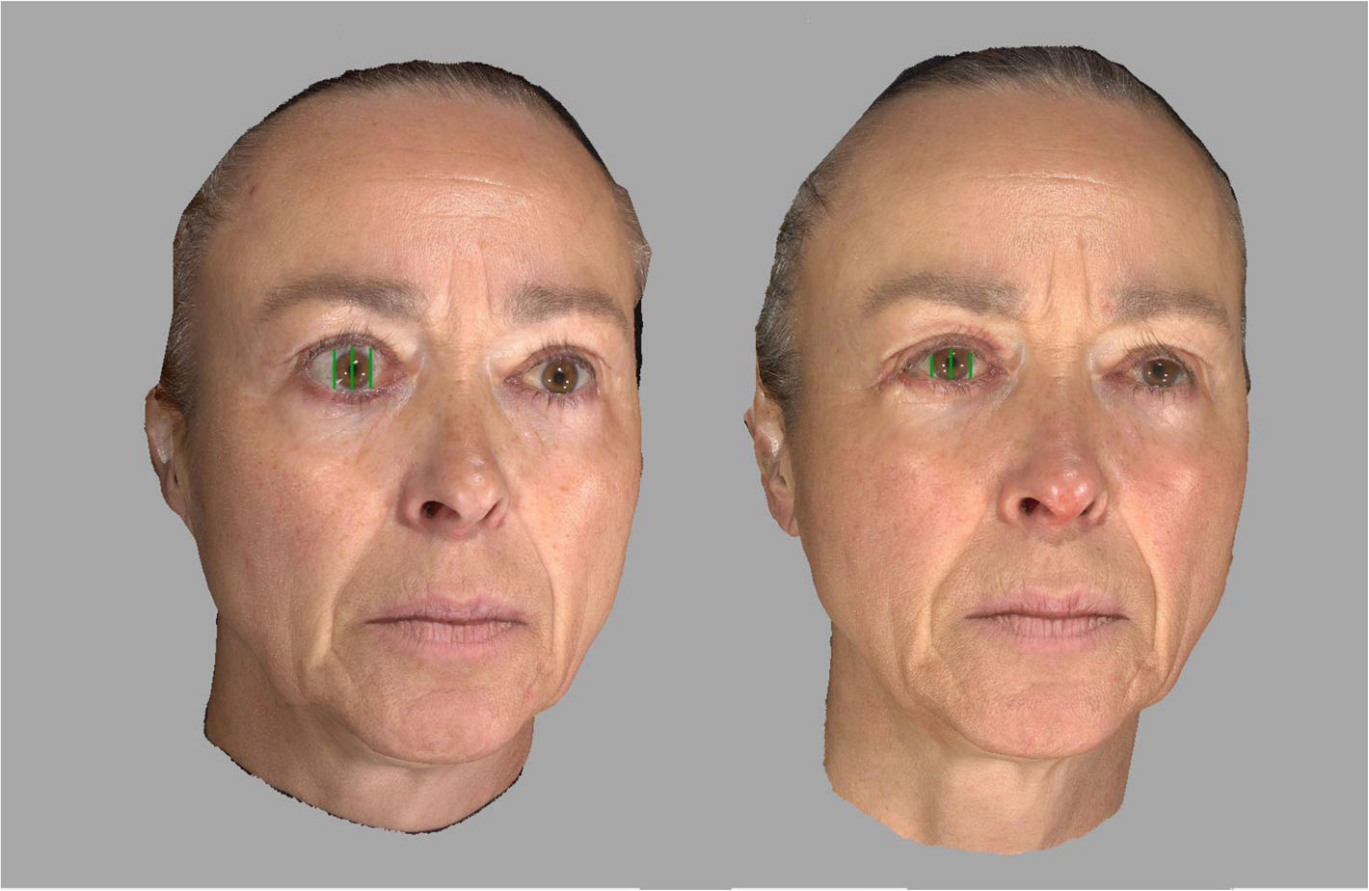
Comparison of presurgical (left) and postsurgical (right) optical scans in a patient after eyelid lengthening on the right site with upper lid retractor recession over a posterior approach and lateral canthoplasty (tarsal strip technique) on the lower lid

The ocular surface area (OSA) and vertical palpebral fissures before and after surgical decompression and after eyelid repositioning and/or after soft tissue refinement were measured digitally using a facial analysis tool (FAT software) as previously described (Fig. 1) [6]. For 3D photography, the Vectra M3 passive stereophotogrammetric system (Canfield Scientific Inc., Fairfield, NJ) was used to obtain detailed three-dimensional static photographs. Additionally, final results after lid refinement surgery and blepharoplasty were evaluated. Furthermore, parallel vertical lines in the pupillary region were created over vertical landmarks that were placed on the upper and lower lids along the central papillary line (CPL), medial papillary line (MPL), and lateral pupillary line (LPL) (Fig. 1). Next, the distances between these landmarks were calculated to objectively monitor changes in the distance of eye opening. They were taken pre- and post-decompression surgery and after adjunctive surgery (Fig. 2). Standard follow-up and 3D examination occurred 6 months after decompression surgery and 24 months after primary and secondary blepharoplasty.

### Planning of the surgical procedure

**Fig. 3:**
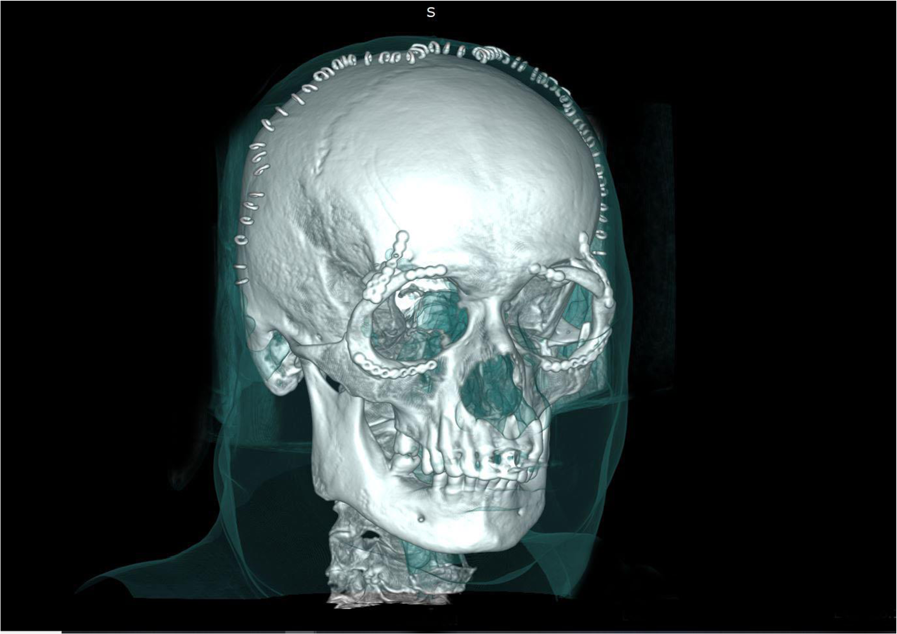
Postoperative CT after 3 wall decompression surgeries with LARA on both sides

Surgical correction of EO is traditionally performed in 4 steps [4]. Most importantly, for optical nerve function and major relief of the orbital protrusion, the process started with decompression surgery:

1) Surgical bony orbital decompression was planned and timed according to the recommendations of the clinical guidelines for the management of endocrine orbitopathy [1, 2]. For orbital decompression surgery, all patients received a preoperative CT scan, and the procedure was planned with iplan (Brainlab software, Brainlab, Feldkirchen, Germany) for navigated surgery. A postoperative CT scan was performed to compare the results, and a follow-up at the ophthalmologist was performed as well. The number of decompressions was indicated by the amount by which proptosis needed to be reduced: < 5 mm required one wall decompression, from 6 to 10 mm required two wall decompressions, and over 10 mm or DON required three wall decompressions. Further considerations for the type of decompression were the presence or absence of advancement of the lateral orbital rim (lateral rim advancement [LARA]) according to Gonzales-Garcia et al. (7), depending on the protrusion of the ocular bulbus (Fig. 3).

2) Extraocular muscle surgery was conducted primarily in cases with persistent diplopia after orbital decompression surgery. The surgical procedure did not affect OSA, because the focus was only on muscular reduction and not orbital soft tissue management. This procedure was performed by ophthalmologists in the tertiary care center.

3) Correction of eyelid retraction (primary blepharoplasty) was performed with the aim of functional eyelid repositioning. Eyelid malpositioning has often been described in EO and mostly affects upper and lower lid retraction [4]. In patients with insufficient lid closure, dry eye syndrome can be prevented. The upper eyelid correction is usually conducted using a levator recession with an anterior or posterior approach. If this is not successful, spacers may be used to lengthen the eyelids. These spacers may be harvested from the nasal septum or ear cartilage. The recession of lower eyelid retractors must often be performed with a spacer to raise the lower lid margin. Further lateral canthal malpositioning may be corrected with canthoplasty or canthopexia.

4) Secondary or esthetic blepharoplasty with removal of excess fat and skin, such as removal of excess fat or skin, was performed according to patient needs. EO likely causes an accumulation of excess fatty tissue around the eyelids, and the skin becomes prominent. The aim of this stage is the removal of this excess tissue with procedures such as standard blepharoplasty to restore a normal appearance to the patient. In addition, a brow lift can be considered in patients with ptosis. The goals of eyelid blepharoplasty are extremely important in patients with dysthyroid ophthalmopathy to create a normal, aesthetic appearance without stigmata. However, in daily practice, extraocular muscle surgery remains rare, and the procedures of primary and secondary blepharoplasty are conducted according to patient needs; thus, the historically described treatment ladder is frequently tailored to the particular situation.

### Statistical analysis

Statistical analysis was performed with SPSS 24.0 (Statistic Package for the Social Sciences; SPSS Inc., Chicago, IL, USA). In the statistical evaluation, we decided to count each orbit separately.

## Results

A total of 65 patients (48 females (73.8%), 17 males (26.2%)) and 120 orbits (110 on both sides (84.6%), 5 right orbits (7.7%), 5 left orbits (7.7%)) with EO were included and analyzed pre- and post-bony decompression and post-blepharoplasty. Adjunctive surgical procedures were performed in 36 patients (55,3%). The mean patient age was 50.6 years (range 30–71). Eleven patients (16.9%) suffered from DON. LARA was performed in 47 cases (94 orbits, 72.3%). The mean follow-up was 6.8 ± 5.6 months after decompression surgery and 24 ± 7 months post-blepharoplasty surgery.

Furthermore, right and left eyes were compared, and statistically significant differences in OSA were only identified preoperatively (2.9 cm^3^ vs. 3.0 cm^3^, *p* = 0.033) but not after decompression surgery (2.4 cm^3^ vs. 2.4 cm^3^, *p* = 0.666) or after blepharoplasty (2.34 cm^3^ vs. 2.27 cm^3^, *p* = 0.215). The preoperative CPL (12.1 mm vs. 12.6 mm, *p* = 0.019) showed statistically significant differences between right and left eyes, which disappeared after decompression surgery (11.0 mm vs. 11.1 mm, *p* = 0.549) and blepharoplasty (10.6 mm vs. 10.4 mm, *p* = 0.250). The preoperative MPL (10.1 mm vs. 10.3 mm, *p* = 0.233) and LPL (11.0 vs. 11.5, *p* = 0.124), post-decompression MPL (9.1 mm vs. 8.9 mm, *p* = 0.495) and LPL (9.5 mm vs. 9.9 mm, *p* = 0.124), and post-blepharoplasty MPL (8.6 mm vs. 8.2 mm, *p* = 0.133) and LPL (9.1 mm vs. 8.9 mm, *p* = 0.337) did not differ between right and left eyes.

The measurement of OSA, as well as the above-mentioned anatomical lines, showed a significant reduction after each surgical step (Table 1). However, the major reduction in OSA (15%) was noted after orbital decompression surgery (2.98 cm^3^ vs. 2.5 cm^3^). After decompression, in 36 patients (55.3%), adjunct therapy became necessary (Table 2). A total of 151 procedures were performed on 144 eyelids. However, a secondary correction in 13 patients (20%), a tertiary correction in 8 patients (12.3%), and a fourth correction session in 5 patients (7,6%) became necessary.

**Table 1:** Parameter changes after orbital decompression surgery and adjunctive surgery. A total of 108 eyes after decompressions surgery were included, as well as *n* = 144 eyelids after adjunctive surgery

Parameter	Preoperative	Post - decompression	*p*-value*	Post - adjunctive surgery	*p*-value*
	(mean ± SD)	(mean ± SD)		(mean ± SD)	
OSA (cm^2^)	2,98 ± 0,85	2,52 ± 0,62	*p* < 0.001	2,31 ± 0,55	*p* = 0.003
MPL (mm)	10,1 ± 2,60	9,35 ± 2,35	*p* < 0.001	8,46 ± 2,14	*p* = 0.005
CPL (mm)	12,4 ± 2,59	10,73 ± 2,19	*P* < 0.001	10,52 ± 1,95	*p* = 0.625
LPL (mm)	11,2 ± 2,73	9,97 ± 2,21	*p* < 0.001	9,0 ± 2,02	*p* = 0.001

*OSA* Ocular surface area, *MPL* Medial pupillary line, *CPL* Central pupillary line, *LPL* Lateral pupillary line; *p Paired t-test, significance set at 0.05

**Table 2:** Characteristics and frequency of patients with adjunctive eyelid surgery (*n* = 36 patients, 117 eyelids, 124 procedures)

	(n) 1st session	(n) 2nd session	(n) 3rd session	(n) 4th session	(n) total
patients	36	13	8	5	
eyelids^a^	75^a^	21^a^	12^a^	9^a^	117^a^
procedures^b^	75^b^	28^b^	12^b^	9^b^	124^b^
**Upper eyelids**^a^	48^a^	10^a^	8^a^	6^a^	72^a^
**indications**					
ptosis	2^a^	4^a^	3^a^	2^a^	11^a^
retraction	15^a^	6^a^	2^a^	1^a^	24^a^
excess tissue	31^a^		1^a^	1^a^	33^a^
contour deficit			2^a^	2^a^	4^a^
**Upper eyelids**	48^b^	10^a^	8^b^	6^b^	72^b^
procedures**					
shortening	2^b^	4^b^	3^b^	2^b^	11^b^
lengthening	15^b^	6^a^	2^b^	1^b^	24^b^
tissue removal	31^b^		1^b^	1^b^	33^b^
lipofilling			2^b^	2^b^	4^b^
**Lower eyelids**^a^	27^a^	11^a^	4^a^	3^a^	45^a^
**indications**					
retraction	15^a^	9^a^	2^a^	2^a^	28^a^
excess tissue	12^a^	2^a^	1^a^		15^a^
scarring			1^a^	1^a^	2^a^
**Lower eyelids**	27^b^	18^b^	4^b^	3^b^	52^b^
**procedures**^b^					
lengthening	15^b^	16^b^	3^b^	3^b^	37^b^
ear cartilage		1^b^			1
mucograft	5^b^	2^b^			7
canthoplasty	6^b^	5^b^	2^b^		13
medpor	2^b^			2^b^	4
lipofilling	1^b^	6^b^			7
scar removal	1^b^	2^b^	1^b^	1^b^	5
tissue removal	12^b^	2^b^	1^b^		15^b^

^a^number of eyelids, ^b^number of procedures

A total of 72 eyelid procedures were performed on the upper eyelid. Tissue removal procedures (33 eyelids) due to excess tissue outnumbered lid lengthening procedures (24 eyelids) due to eyelid retraction, and lid lengthening procedures (36 eyelids) due to eyelid retraction were similarly distributed. In the lower lid region, 52 procedures were performed on a total of 45 eyelids. Lid lengthening procedures (37 performed on 28 eyelids) due to retraction outnumbered tissue removal procedures (15 performed on 15 eyelids). Other procedures (e.g., brow suspension, plate removal) were carried out on 27 eyelids. Interestingly, procedures on the lower eyelid were more complex and frequently required grafts for tissue suspension. Procedures such as cartilage grafting (*p* = 2), mucosal grafts (*p* = 7), and lipofilling (*p* = 7) were more frequent in lower lid surgery. Similarly, canthoplasties (*p* = 13) were more likely to be conducted in lower lid surgery (Table 2).

Other surgical procedures, e.g., brow lift (*n* = 6) or plate removal (*n* = 2), are not listed in Table 2, because the surgical result had no effect on OSA.

## Discussion

Dysthyroid optic neuropathy (DON) is the worst form of EO and the primary target of surgical treatment. The general aim of treatment in patients with endocrine orbitopathy is to achieve optimal functional and psychosocial rehabilitation. For immediate release of intraorbital pressure, orbital wall decompression surgery is performed. We can show that it also has a major effect on the reduction in OSA. In the study cohort, there was a decrease of 15% (0,5 cm^2^), which is comparable to previous findings in a Korean population [7]. Park et al. described a decrease of 13% (from 190.5 to 165.5 mm^2^), which was also significant. However, the overall OSA was lower in the Asian population, which was probably caused by ethnic differences. Thus, it might be difficult to compare an Asian with a Caucasian population.

We chose a modification of the classical four-step approach for surgical treatment of EO as suggested by Shorr and Seiff [4]. If the same eyelid was not affected by more than one pathological condition, step 3 (eyelid elongation) was performed in the same procedure as step 4 (excessive tissue removal), thus sparing the patient from additional procedures under general anesthesia. However, recently, Bernardini and coworkers described a single-stage surgical procedure in a 40-patient multicenter cohort [8]. They performed all procedures during the initial surgery and claimed advantages in patient satisfaction and reduction in health care costs. Although the patients potentially prefer fewer surgical steps, based on the authors’ experience, the results of primary decompression surgery need some time to settle. Proptosis and the required tissue and/or tissue excess are hard to determine during the first surgery. Furthermore, in the case of secondary or tertiary corrections, scarring is likely to occur, thus making the result even more unpredictable [8].

Wu et al. described factors leading to a greater reduction in proptosis, including larger preoperative proptosis, balanced decompression, EO duration < 4 years, and history of orbital radiation. However, OSA and anatomical landmarks were not measured in this study, and thus, its direct effect is still not entirely clear. However, it is likely that by reducing proptosis, OSA will also be reduced, and the above-mentioned parameters should be considered prior to surgical treatment [9].

In our patient population, there was a difference in procedures performed on the upper and lower lids. Upper lid procedures were more likely to remove excess tissue, whereas lower lid procedures were more focused on lid lengthening. Most frequently, cartilage graft, mucograft, and medpor® (porous polyethylene implants, Stryker, Kalamazzo, USA) were used for lengthening procedures. In a literature review, Ribeiro et al. reported that evidence-based data are still lacking [10]. From the authors’ perspective, the appropriate indications for lid lengthening procedures or blepharoplasty are essential and will, as the findings of our study present, significantly alter OSA. Nonetheless, in lower lid operations, surgeons should be aware that there is likely a lack of tissue after healing, and thus, resection of excess skin and fat in this area should be performed with caution. Although the above-mentioned procedures for lid lengthening were not frequent enough in our population to conclusively determine a statistical difference, the authors agree with Hayashi and coworkers, who favored the use of autologous tissue (cartilage) for lid lengthening in a population with lagophthalmus [11].

Golan et al. reported the retraction of the upper eyelid in 85% of patients [12]. However, in our study cohort, the number was lower (20, 8%). These differences might be attributable to different surgical approaches, ethnic background, or disease severity. However, the actual reasons remain unclear, and the stated explanations for these differences are hypothetical. The measurement of OSA revealed that it was significantly reduced after each surgical step and also after lid refinement, which indicates that the procedure is performed not only for the aesthetic perception of patients but also for functional outcomes. This highlights the necessity of adjunct procedures after initial decompression surgery to achieve a satisfying outcome. Increased OSA may lead to dry eye syndrome and accompanying problems. Interestingly, the preoperative difference in OSA between the right and left eyes was eliminated after surgical treatment.

### Bias and limitations

Although the study cohort represents, to the best authors’ knowledge, the largest population of Caucasian patients with EO, the total number is too small for a robust statistical evaluation. However, the results show that there are significant differences in surgical procedures addressing the upper and lower lids, which should be considered prior to treatment.

## Conclusion

Adjunctive surgical procedures addressing the upper and lower lids have a great impact on the resulting ocular surface area. The procedures should be tailored according to patient needs and should be carefully evaluated for each lid independently.

## Data Availability

All data can be shared.
